# Executive function and self-regulated exergaming adherence among older adults

**DOI:** 10.3389/fnhum.2014.00989

**Published:** 2014-12-05

**Authors:** Cay Anderson-Hanley, Paul J. Arciero, Nicole Barcelos, Joseph Nimon, Tracey Rocha, Marisa Thurin, Molly Maloney

**Affiliations:** ^1^Healthy Aging and Neuropsychology Lab, Department of Psychology, Union CollegeSchenectady, NY, USA; ^2^Human Nutrition and Metabolism Laboratory, Health and Exercise Sciences Department, Skidmore CollegeSaratoga Springs, NY, USA; ^3^The Division of Counseling Psychology, Department of Educational and Counseling Psychology, School of Education, University at AlbanyAlbany, NY, USA

**Keywords:** exergame, aerobic exercise, cognition, older adults, self-regulation, executive function

## Abstract

The rise in dementia and the evidence of cognitive benefits of exercise for the older adult population together make salient the research into variables affecting cognitive benefit and exercise behavior. One promising avenue for increasing exercise participation has been the introduction of exergaming, a type of exercise that works in combination with virtual reality to enhance both the exercise experience and health outcomes. Past research has revealed that executive function (EF) was related to greater use of self-regulatory strategies, which in turn was related to greater adherence to exercise following an intervention (McAuley et al., 2011). Best et al. (2014) found improvement in EF related to adherence to exercise post- intervention. Anderson-Hanley et al. (2012) found that for older adults aerobic exergaming yielded greater cognitive benefit than traditional exercise alone; however, questions remain as to the possible impact of greater cognitive benefit and other factors on participants’ involvement in exercise following the end of an intervention. The current study presents follow-up data exploring the relationship between EF, self-regulation, and exercise behavior in the post-intervention (naturalistic) period. Herein, it was predicted that higher EF at the start of the naturalistic window, would predict subsequent exercise with an exergame. Contrary to expectations, results suggest that those with poorer EF are likely to exergame more frequently. The results of this study contradict previous literature, but suggest an interesting relationship between EF, self-regulation, and exercise behaviors when exergaming is employed, particularly with older adults with some cognitive decline. We hypothesize that other factors may be at work, perhaps expectation of cognitive benefit might act as a unique motivator.

## Introduction

The positive impact of exercise on cognition has been well documented in the scientific literature (Kramer et al., 1999; Colcombe et al., 2004; Hogan et al., 2013). Longitudinal research in Europe indicated that people aged 50 years and older who participated in any kind of physical activity showed less cognitive decline after 2.5 years than those who performed no physical activity (Aichberger et al., 2010). Randomized controlled trials (RCTs) and exercise intervention studies, moreover, have revealed that both aerobic exercise (Colcombe and Kramer, 2003; Smith et al., 2010) and resistance training (Cassilhas et al., 2007; Liu-Ambrose et al., 2010; Nagamatsu et al., 2012) improve cognition, especially aspects of executive function (EF) including planning, decision making, and inhibition. Nonetheless, research reveals that only 4% of adults exercise at the recommended frequency and intensity (Gordon-Larsen et al., 2004).

Given the well-established evidence-base suggesting that exercise offers cognitive benefit for the older adult population in particular (Colcombe et al., 2004; Hindin and Zelinski, 2012), and the concomitant rise in rates of dementia (Steffens et al., 2009), research efforts must now turn to understanding variables which may predict this populations’ participation in and adherence to a fitness regimen. An understanding of the variables that predict involvement in exercise behavior (both during and after prescribed interventions) could lead to more targeted interventions for older adults and, therefore, to more exercise and cognitive benefit.

Various theoretical approaches have evolved that attempt to understand behavioral constructs and related variables that may moderate the relationship between exercise intervention and maintenance of exercise behavior. While our research on the cognitive benefits of exercise for older adults led us to examine factors related to adherence within that context, it is important to note that significant contributions to the work on the role of EF in exercise adherence has resulted from a broader set of research. That body of literature has pioneered the role of EF in self-regulation and examined that relationship in diverse topics ranging from health behaviors and consumer research on decision making, lending further support to the notion that EF plays a critical role in the maintenance of desirable behaviors (e.g., in treatment of substance abuse, in pain management, recovery from brain injury; Solberg Nes et al., 2009; Hofmann et al., 2008; Hunt et al., 2013; Bickel et al., 2014). Generally, EF is the mechanism that allows a person to make choices about behaviors that may not immediately positively affect them, but will have positive effects in the long run (e.g., suppressing an unconsidered impulse to buy something expensive, going to the gym daily to make incremental steps toward health). Executive function requires a host of underlying cognitive, biological, and social processes to function (Hall and Fong, 2007). It is hypothesized that EF may play a major role in various behaviors, but especially self-motivated exercise behaviors, because it is implicated in the delay of gratification and participation in activities which may not yield immediate external reward (Hall and Fong, 2007).

One approach, the so-called social-cognitive approach, is undergirded and perpetuated by findings that variables like self-efficacy and social support (Chogahara et al., 1998; McAuley and Blissmer, 2000) may be mechanisms through which exercise behaviors occur in the absence of other extrinsic motivations (e.g., rewards for participating in employer health and wellness program). A variant of those theories cited in the social and health psychology literature, the Theory of Planned Behavior (TBP), has been suggested to play a role in health-related behaviors (Godin and Kok, 1996). The theory attempts to explain how people’s attitudes and beliefs about objects and themselves influence their participation in health-relevant behaviors (Ajzen, 1985). Self-efficacy is a core control belief in the TBP model and has been robustly demonstrated to play a role in people’s engagement in exercise behaviors.

Another approach to analyzing maintenance of exercise behavior post-exercise intervention is a more biological approach that has been motivated by existent evidence suggesting that exercise structurally and functionally alters the brain (Colcombe et al., 2006; Hillman et al., 2008). Research from this neurocognitive approach focuses, in particular, on the role of top-down processes, including EF in adherence to exercise behaviors. The temporal self-regulation theory (TST), a theory of the neurocognitive approach, provides a theoretical framework through which exercise intervention, EF, and regimen adherence may relate. Generally, TST offers an explanation for how people decide to engage in immediate behaviors that yield later, but not immediate, effects. According to TST, there are many underlying social, biological, and cognitive processes that work together to enable a person to engage in behaviors that will produce delayed benefits; these processes are crucial for the motivation required to continue a regular exercise regimen (Hall and Fong, 2007).

In a recent RCT and subsequent follow-up study of these phenomena, Best et al. (2014) examined the relationship between EF and adherence to an exercise routine post-fitness intervention. The researchers hypothesized that increased EF (due to an exercise intervention) would predict continued participation in exercise once the intervention ceased. Results revealed that women who had experienced greater EF benefit from the exercise intervention were more likely to adhere to an exercise routine in the year following the cessation of the intervention. These results were unaffected by any potential covariates (i.e., age, education, depression, and MOCA scores). Best et al. (2014) research suggests that functional changes in brain processes, especially EF, may subsequently impact maintenance of exercise behavior.

McAuley et al. (2011) conducted a year-long exercise intervention with a sample of older adults and subsequently analyzed exercise behaviors in the year following the intervention. Expanding on prior research and connecting aspects of the social-cognitive approach with the neurocognitive approach, they included various measures of EF, such as working memory, inhibition, and task switching, while also measuring the use of self-regulatory strategies and self-efficacy. The results revealed that specific types of EF (i.e., task switching and inhibition) were related to greater use of self-regulatory strategies, which in turn was related to higher self-efficacy; this higher self-efficacy predicted greater adherence to an exercise routine in the year following the cessation of the exercise intervention. McAuley et al. (2011) research is especially important for understanding health-related behaviors, because it suggests that both social/personal characteristics and neurocognitive processes play a role in the maintenance of healthful behavior, such that self-efficacy may mediate elements of EF to influence exercise behavior.

The current research aims to expand on research such as Best et al. (2014) and McAuley et al. (2011) by examining how change in EF relates to and plays an important role in exercise adherence. The question investigated in the current research is whether or not EF may be related to people’s participation in an exercise behavior; in particular, after the conclusion of a prescriptive exercise intervention period. In our previous research we examined the impact of exergaming (“cybercycling”^1^) on cognitive functions in older adults (Anderson-Hanley et al., 2012). Exergaming is a type of exercise that works in combination with virtual reality and/or videogames to enhance both the exercise experience and possibly also relevant mental processes (e.g., Wii Fit and X-Box Kinect) and is a promising avenue for increasing exercise participation. We found that exergaming yielded greater cognitive benefit, specifically EF, than riding a stationary bike alone (despite a similar dose of exercise in terms of miles, minutes, and intensity). Crucial questions remain, however, as to the possible impact of such a cognitive benefit and other relevant factors on participants’ involvement in exercise behaviors following the end of the exergaming intervention. The current research is a follow-up study which further explores the relationship between exercise behavior, self-regulation and executive control in the post-intervention period of the above noted cybercycle study.

### Hypothesis

As demonstrated by prior research (McAuley et al., 2011; Best et al., 2014), it was predicted that EF at the start of the naturalistic exercise window (thus at the end of the cybercycle randomized trial), would predict frequency of exercise on the cybercycle post-intervention.

## Methods

### Sample characteristics

In a previous randomized clinical trial, 102 older adults were recruited from local independent living facilities and randomized to one of two exercise conditions: physical exercise alone (traditional stationary bike) vs. interactive virtual reality-enhanced stationary cycling (cybercycle). Individuals with known neurological disorders (e.g., Alzheimer’s disease) or significant functional disabilities that would restrict participation in physical exercise or cognitive testing were excluded from participation; written physician approval to exercise was required. The study was approved by a human subjects review committee and written informed consent was obtained for all enrollees; the study was registered as a national clinical trial^2^ (NCT01167400). Sixty-three participants completed the 3-month randomized portion of the study (results reported elsewhere; see Anderson-Hanley et al., 2012), and 51 were willing and able to continue exercising during the subsequent naturalistic exercise period and reported for final testing after that 6-month window. The average age of the group that participated during the naturalistic window (*n* = 51) was 79.0 years (SD = 8.4) and their average years of education was 13.7 (SD = 2.5); 71% were women and the sample was predominantly Caucasian (>90%). Data from 30 participants were in the present analyses; they include those who had also completed the RCT, completed evaluations at the end of the naturalistic exercise window, had data on the variables of interest herein, and had valid exercise data (those with zero ride frequency who had a physical injury or had moved away were dropped from the analyses). Outliers were also examined and consistent with Best et al. (2014), results are also presented with one outlier removed.

### Procedures

During the RCT, participants were randomly assigned to either the traditional stationary bike group or the cybercycle group. Participants in both groups rode the same recumbent stationary bike, but in the cybercycle condition a virtual reality screen was active and participants interacted with a virtual bike tour, steering and competing with avatars. All participants were encouraged to gradually increase their total time of exercise to 45 min, 5 days a week in their respective condition, consistent with American College of Sport Medicine (ACSM) standards (Garber et al., 2011). Participants were instructed to adhere to their assigned exercise condition for 3 months. After each exercise session participants documented their ride behaviors on a paper log. Ride logs were collected from the study sites and used to calculate ride statistics (frequency, intensity, duration). The average number of rides for the cybercycle group and control groups were 51.3 (SD = 3.32) and 53.3 (SD = 3.14), with average durations of 35.5 min and 33.8 min, respectively. After 3 months in their randomized conditions, participants were invited to engage in naturalistic exercise for 6 months.

During the naturalistic exercise window, participants in both the control and cybercycle conditions were encouraged to exercise 5 days a week for 45 min per session while using the cybercycle in its fullest capacity (e.g., navigating various bike tours or playing a dragon chase videogame). It is the naturalistic exercise window that is the focus of this report. Again, paper ride logs were collected from the study sites and used to calculate ride statistics as noted above. We chose to focus on the middle 3 months of the 6-month ride window due to missing data from some sites at the beginning and end of the naturalistic window. Using a 3-month window also provided a comparable exercise window to that studied in the RCT.

### Measures

Tests of cognitive functioning were administered at enrollment (baseline), 1 month after that (pre-intervention), and then again after the 3-month randomized exercise conditions (post-intervention/pre-naturalistic exercise window). The current research examines the relationship between EF post-intervention and exercise behavior during the follow-up naturalistic window, during which participants were encouraged to utilize the cybercycle in its full capacity.

#### Executive function

The original study utilized a full battery of neuropsychological tests to characterize the sample and track changes; alternate forms were used at each time point. Consistent with recent data published on this topic (Best et al., 2014), the current analysis focused on the Stroop Test (Stroop, 1935). The Stroop Test has proven to be one of the most effective measures of EF as the brain areas activated during this task are associated with cost-benefit decision making and impulse control (Strauss et al., 2006). A shortened, 40-item version of the Stroop was used, presenting colored blocks first (Stroop A), followed by black text color names (Stroop B), and finally color names printed in contrasting colored ink (Stroop C; adapted from Van der Elst et al., 2006). In an effort to partially replicate the recently reported work of Best et al. (2014), Stroop C—Stroop A was used as a measure of EF, and it was the change in this EF variable over the course of the initial RCT that was the focus of our analyses. Given that that time in seconds is the outcome, greater Stroop C-A scores indicate worse EF; thus, a negative change score (post-RCT minus pre-RCT), indicates improving EF.

#### Exercise attitudes measures

In addition to cognition, other variables were measured to examine possible influences on exercise behaviors; in particular: self- efficacy, perceived benefits and barriers to physical activity, and attitudes and motivations towards physical activity and the cybercycle specifically. Self-efficacy was measured using the Self Efficacy for Physical Activity Survey (SEPAS; Sallis et al., 1988); questions assessed how confident participants were that they could complete exercise behavior in the face of various barriers such as needing to wake up early, set aside time, and continue adhering to a routine during stressful times. The Exercise Benefits/Barriers Scale (EBBS; Sechrist et al., 1987) was used to measure the degree to which participants saw both benefits and barriers in exercise. Questions assessed whether participants thought exercise would benefit variables such as their physical and mental health and whether participants perceived a number of barriers, such as embarrassment, in adhering to an exercise routine. Lower scores indicate greater endorsement. The Social Support and Exercise Survey (SSES; Sallis et al., 1987) was used to measure the degree to which participants received support from family members and friends for exercise endeavors. Participants’ motivations for exercising in general and for completing the cybercycle study, specifically, were assessed. The Motives for Physical Activities Measure—Revised (MPAM-R; Ryan et al., 1997) was used to evaluate general exercise motivations. Participants rated the degree to which they were exercising for various reasons including wanting to be fit, look better, stay in shape, and because others motivating them.

#### Motivation to exercise for cognitive benefit

Given unexpected findings noted below, we probed our dataset for items that tapped participant motivation to exercise for cognitive benefit (MECB). Two items were identified that appeared to capture this construct. One item was taken from the Cybercycling Attitudes Test (CAT), which was developed by Nimon et al. (2009) to measure attitudes towards the cybercycle intervention: “*Exercising on the cybercycle will make me think more clearly*” (Likert scale 0–5, disagree to agree). The other item (#34) was taken from the EBBS: “*Exercising increases my mental alertness*” (Likert scale 1–4, agree to disagree). The EBBS item was reversed scored and scaled to match the CAT. These items were found to be somewhat correlated (*r* = 0.33) and summed to form a two-item measure of MECB.

#### Other measures

Participants were characterized as meeting criteria for mild cognitive impairment (MCI), if they scored −1.5 SD on three domains in the RCT’s comprehensive neuropsychological battery (Jak et al., 2009). Participants’ health was assessed using the Pennebaker Inventory of Limbic Languidness (the PILL) to assess participants’ experience of common health symptoms (PILL; Pennebaker, 1982). The PILL assesses how often people have recently experienced various common symptoms and sensations, such as swollen joints, asthma, and dizziness.

### Statistical analyses

Partial correlations were conducted in SPSS version 19 (IBM Corporation, 2012). Descriptive statistics were computed in EXCEL (Microsoft Corporation, 2010).

## Results

### Preliminary results

The current sample (*n* = 30) was compared with those who completed the original RCT but did not participate in the naturalistic window (*n* = 33). No significant differences were found on key variables (i.e., age, education, MCI status, EF) between the sample herein of those continuing to be followed in the study after the RCT and those from the original sample who did not continue on to participate in the naturalistic exercise window. Descriptive data is presented in Table 1.

**Table 1:** **Demographics of current sample compared with the non-continuing sample**.

	Current sample^a^			Non-continuing sample^b^			
	ave	SD	*n*	ave	SD	*n*	*p*
**Demographics**
group (% cybercycle group)	60%		30	36%		33	0.06
age	79.5	9.2	30	78.5	8.5	33	0.64
education (yrs)	14.1	3.2	30	13.6	1.9	33	0.45
sex (% female)	67%		30	79%		33	0.29
MCI classification	40%		30	21%		33	0.11
**Exercise outcome**
ride frequency	21.0	20.2	30	27.8	25.8	23	0.29
**Executive function**
Stroop C-A	29.24	14.6	30	29.71	23.7	31	0.93
Stroop C-A (prior intervention change: post-pre)^c^	−3.99	0.32	30	0.32	12.02	29	0.17
**Exercise attitudes**
Exercise benefits and barriers scale (benefits)	61.9	7.2	30	59.7	13.6	26	0.43
Exercise benefits and barriers scale (barriers)	35.7	7.6	30	39.54	8.5	26	0.08
Social support for exercise scale (family)	22.8	12.6	30	23.1	10.2	25	0.93
Social support for exercise scale (friends)	24.6	11.3	30	23.9	11.1	22	0.83
Self-efficacy physical activity scale (total)	46.3	9.4	30	46.5	10.3	31	0.94
Motivation for physical activity measure (enjoy)	35.3	9.3	30	33.1	12.0	30	0.43
Motivation for physical activity measure (appearance)	29.4	7.7	30	27.8	9.8	30	0.48
Motivation for physical activity measure (fitness)	32.0	4.2	30	30.2	5.8	30	0.17
Motivation to exercise for cognitive benefit	9.0	1.4	30	8.1	2.9	32	0.14

*Note: Data on measured variables were collected post-randomized exercise/pre-naturalistic exercise*.

*^a^The “Current Sample” refers to those who completed the original RCT and the follow-up (even if they had zero rides)*.

*^b^The “Non-continuing Sample” refers to those who completed the original RCT, but not the follow-up*.

*^c^Used change in difference score formula consistent with Best et al. (2014)*.

### Predictors of exercise adherence

Partial correlations (list-wise) were conducted to examine the relationship between exercise frequency (number of rides on the cybercycle based on participants ride logs; average duration = 35.7 min and average intensity = 130 kcal) and change in EF (formula above adapted from Best et al., 2014), as well as exercise attitudes (benefits, barriers, social support, self-efficacy, and motivations), other possible predictors (MCI status, physical illness), all while controlling for randomized group assignment, age, education, and baseline EF.

Initial analyses including all eligible cases (*n* = 30) revealed a trend-level negative correlation between EF improvement (during prior RCT) and subsequent exercise frequency; indicating, that contrary to expectations, those with declining EF at the start of the naturalistic window went on to exercise more frequently than those who had made EF gains (*r* = 0.38; *p* = 0.0549). As noted above, consistent with Best et al. (2014) approach of excluding outliers, one extreme outlier was identified and removed from analyses. The reanalysis confirmed a strong inverse relationship between prior EF decline and later exercise adherence (*r* = 0.43; *p* = 0.03). Partial correlation results are reported in Table 2.

**Table 2:** **Partial correlations with ride frequency**.

	Ride frequency
	Sample post-RCT^a^ (*n* = 30)		Excluding outlier (*n* = 29)	
	*r*	*p*	*r*	*p*
**Demographics**
MCI classification	0.18	*0.38*	0.13	*0.54*
Physical illness scale	0.08	*0.69*	0.06	*0.77*
**Executive function**
Stroop C-A (prior intervention change: post-pre)^b^	0.38	***0.05***	0.43	***0.03***
**Exercise attitudes**
Exercise benefits and barriers scale (benefits)	−0.28	*0.16*	−0.15	*0.47*
Exercise benefits and barriers scale (barriers)	0.37	*0.06*	0.37	*0.07*
Social support for exercise scale (family)	−0.10	*0.62*	−0.11	*0.59*
Social support for exercise scale (friends)	0.03	*0.89*	−0.05	*0.81*
Self-efficacy physical activity scale (total)	0.52	***0.01***	0.42	***0.04***
Motivation for physical activity measure (enjoy)	−0.15	*0.48*	−0.20	*0.33*
Motivation for physical activity measure (appearance)	−0.41	***0.04***	−0.55	***0.004***
Motivation for physical activity measure (fitness)	0.00	*1.00*	0.01	*0.98*
Motivation to exercise for cognitive benefit	0.35	*0.08*	0.32	*0.12*

*Note. Control variables included: group, age, education, and prior executive function (at enrollment)*.

*^a^The “Sample post-RCT” refers to those who completed the original RCT and the follow-up (even if they had zero rides)*.

*^b^Used change in difference score formula consistent with Best et al. (2014)*.

Because of the above noted unexpected inverse relationship between EF improvement and exercise adherence, we considered the possibility that our sample might differ from typical exercise studies in that the intervention was novel (combining interactive mental and physical exercise), and participants had received information regarding the possible cognitive benefits of exercise. Furthermore, since our sample was older than those in the study by Best et al. (2014) and many other typical exercise RCTs, we did have a greater proportion that was already starting to experience some cognitive decline and might be thus more motivated to try to improve their function and slow decline. We wanted to evaluate the possible impact of participants’ motivation to exercise due to this unique feature of the RCT intervention and reviewed our data set for specific items that tapped participant motivation to exercise for possible cognitive benefits (see items identified and selected under Section Measures above). Motivation to exercise for cognitive benefit was thus also evaluated for its relationship to outcomes, and while statistically non-significant, indicates a possible trend (*r* = 0.35; *p* = 0.08).

A significant relationship between self-efficacy for physical activity and ride frequency (*r* = 0.52; *p* = 0.01) was also seen, in that individuals with higher exercise self-efficacy were found to exercise more frequently during the naturalistic window compared to those with lower personal efficacy for physical activity. This finding is in line with TPB and supports previous findings (Ajzen, 1985; McAuley and Blissmer, 2000; McAuley et al., 2011). Additionally, a significant inverse relationship was found between motivation to exercise for physical appearance and exercise adherence (*r* = −0.41, *p* = 0.04).

## Discussion

Results of a small sample of older adults exercising following a 3-month randomized trial of exergaming vs. traditional exercise, suggests a somewhat curious and unexpected relationship between EF and self-regulated engagement in exergaming. The relationship found herein suggests an inverse relationship between EF improvement and the frequency of exergaming, which contradicts previous literature on the effects of EF on exercise adherence via increased self-regulation (McAuley et al., 2011; Best et al., 2014). Among this particular sample of older adults, decline in EF during a prior exercise intervention was associated with greater exercise frequency during a follow-up window.

One hypothesis to explain this apparent inverse relationship is that given the nature of this sample (older than some exercise trial samples) and the specific type of exercise involved (i.e., exergaming, involving interactive mental and physical exercise), the exergaming may have been more motivating for participants who were already experiencing some cognitive decline as they may have been hopeful it would yield cognitive benefit. This would fit with TST, as participants experiencing some cognitive decline might be more willing to engage immediate exercise given the possibility that they might yield cognitive and physical health benefits eventually. However, our examination of items related to MECB did not yield a statistically significant relationship, but a possible trend in the expected direction with the outcome of exercise behavior (cybercycle rides). It may be that our *post hoc* generated measure of motivation was not sufficient to capture this factor or that of other factors may be more important in determining the relationship between EF and exercise behavior. Physical appearance was the only motivation item that produced a significant relationship with exercise behavior, in that motivation to exercise for physical appearance was inversely related to exercise adherence. Withall et al. (2011) found a similar finding in their examination of individuals’ motivations for engaging in an organized physical activity. One hypothesis for their finding is that those who indicated they were motivated to exercise for physical appearance may have dropped out if they did not see immediate improvements in their appearance. Additionally, previous studies which have found motivation to exercise for physical appearance to decrease with age (Trujillo et al., 2004; Dacey et al., 2008), suggesting that our population is likely more motivated by other factors. In keeping with TST, it may be that participants were more motivated to exercise due to intrinsic factors, rather than extrinsic reasons. Future research is needed to examine the specific relationship between EF, self-regulation, and exercise behaviors when exergaming is employed. It may be important to develop and measure motivation for exercise to benefit cognition, and to specifically focus on the relationship among these variables when targeting exercise interventions for older adults with some cognitive impairment.

Another possible explanation for the unexpected inverse relationship between EF and exercise behavior is that those who did not exercise on the cybercycle during the naturalistic exercise period may have engaged in other physical activities, such as walking on a treadmill. This was not prospectively assessed and is a limitation of this study; rather we depended on participants to sustain the expectation of “no changes to current physical activity” as stated at the beginning of the study. However, a few participants noted in the bike logs that they had participated in other physical activity (e.g., treadmill time). Future research should prospectively and specifically track other physical activity.

Another consideration in interpreting these findings is that family members and caregivers of older adults experiencing some cognitive decline may serve as external motivators, changing self-regulation into other-regulation. For example, a spouse may be concerned that their loved one is not as mentally sharp and fears further decline into dementia, so they may increase their own efforts to facilitate a participant showing up for their allotted exercise slot on the cybercycle. Although we did assess social support for exercise, it is unclear whether some participants would have been able to report this in a way that would capture the possibly subtle, but substantial impact of instrumental assistance some caregivers may have been providing. Future research might find a way to clarify this possible impact on regulation by extension.

Furthermore, it is possible that these results do not entirely contradict the recent report of Best et al. (2014), but rather may illuminate a more complex relationship between changing cognition across later life and exercise behaviors. The sample in their study was comprised of women nearly 70 years old with normative performance on a screen for MCI, whereas our sample was on average a decade older and more impaired overall (40% met criteria for MCI). Thus, some in our sample would have been slipping cognitively regardless of intervention, and while exercise may not have “improved” EF in an absolute way for some, it may have slowed decline (e.g., Lautenshlager et al., 2008). Our sample may have overlapped with Best’s sample, including some normative participants for whom a positive linear relationship with exercise adherence could apply; however, result may be clouded by the mix of more impaired participants for whom may exist a different relationship between EF and adherence. A larger sample, perhaps also a matching sample of normative and MCI participants, would be needed to discern the possible merits or lack thereof of this speculation.

### Strengths

These participants engaged previously in two randomized exercise conditions that provided an interesting comparison since they both involved the same aerobic exercise, but varied mental interactivity. Other studies may assign participants to groups that involve different forms of exercise that may not be comparable on a key variable such as HR (Best et al., 2014), while others may compare an older adult group that is engaging in exercise and a group that is engaging in a non-physical activity (Evers et al., 2011). Participants in the present research engaged in the same type of physical activity during the RCT, thus providing an equivalent starting point in terms of recent prior physical activity; that is, no group was inadvertently advantaged/disadvantaged by having to “catch up” on aerobic exercise, which could have affected results. Instead, during the naturalistic exercise window studied herein, all participants were free to immerse themselves in a virtual tour while engaging aerobic exercise to which their body would have already become accustomed.

Unlike some previous research, there was no transportation barrier for these participants. The equipment and all necessary materials were available in the participant’s residences for ease of access. This greatly reduced the amount of effort required of participants which has been found to deter exercising in previous studies, such as bad weather or having to take public transportation (Evers et al., 2011).

### Limitations

A major limitation of this study was the impact of randomized experience in the control (bike only) and experimental group (cybercycle). By giving the participants the freedom to choose which way they would like to use the bike after the initial 3-month intervention period, those participants that were on the stationary bike seem to have engaged in the cybercycle more frequently, perhaps in part due to its novelty. Those who had been originally assigned to the cybercycle may have lost interest after having been required to exercise on it for 3 months, thus either discontinuing or perhaps choosing different (unspecified/non-bike) forms of exercise. As noted above, this limitation should be addressed in future research by prospective assessment of other forms of physical activity. Other limitations include the small sample size and the variability of the sample (some clearly normative older adults, with some starting to experience cognitive decline). The use of multiple statistical tests also may have led to escalating Type I error rates.

### Future research

Future research might be able to further explore these preliminary findings by examining a larger sample of older adults with some cognitive decline present, and carefully measuring EF, self- and other-regulation, exercise behaviors (as targeted, but also with consideration of non-compliance), and if examining a novel form of exercise, such as exergaming, consider measuring specific variables that could be salient (such as MECB). A larger sample would allow more sophisticated analysis of mediation and moderator models, as well as consideration of change over time that was precluded here by a small sample.

## Conclusion

This report presents preliminary evidence of an unexpected inverse relationship between EF and exercise adherence among older adults. It was hypothesized that that this relationship might be explained by some cognitive decline present in the sample and increased motivation to exercise to protect cognitive function. The latter being an outcome perceived to be possible via the unique interactive mental and physical exercise offered in the exergame (virtual reality stationary bike) utilized herein. Further research is warranted with a larger sample, enabling comparison of possible subgroups (e.g., normative vs. MCI), and examining additional factors affecting motivation and adherence (e.g., exercising for cognitive benefit and/or the possible instrumental role of caregivers in adherence).

## Conflict of interest statement

The authors declare that the research was conducted in the absence of any commercial or financial relationships that could be construed as a potential conflict of interest.

## Footnotes

^1^stationary bike with a virtual reality screen that displays interactive bike tours with on-screen competition or a videogame.

^2^clinicaltrials.gov
